# Alkaloids Induce Programmed Cell Death in Bloodstream Forms of Trypanosomes (*Trypanosoma b. brucei*)

**DOI:** 10.3390/molecules13102462

**Published:** 2008-10-03

**Authors:** Vera Rosenkranz, Michael Wink

**Affiliations:** Heidelberg University, Institute of Pharmacy and Molecular Biotechnology. INF 364, 69120 Heidelberg, Germany

**Keywords:** Trypanosomiasis, leukemia, *Trypanosoma brucei*, Jurkat APO-S, alkaloids, programmed cell death

## Abstract

The potential induction of a programmed cell death (PCD) in *Trypanosoma b. brucei* by 55 alkaloids of the quinoline, quinolizidine, isoquinoline, indole, terpene, tropane, steroid, and piperidine type was studied by measuring DNA fragmentation and changes in mitochondrial membrane potential. For comparison, the induction of apoptosis by the same alkaloids in human leukemia cells (Jurkat APO-S) was tested. Several alkaloids of the isoquinoline, quinoline, indole and steroidal type (berberine, chelerythrine, emetine, sanguinarine, quinine, ajmalicine, ergotamine, harmine, vinblastine, vincristine, colchicine, chaconine, demissidine and veratridine) induced programmed cell death, whereas quinolizidine, tropane, terpene and piperidine alkaloids were mostly inactive. Effective PCD induction (EC_50_ below 10 µM) was caused in *T. brucei* by chelerythrine, emetine, sanguinarine, and chaconine. The active alkaloids can be characterized by their general property to inhibit protein biosynthesis, to intercalate DNA, to disturb membrane fluidity or to inhibit microtubule formation.

## Introduction

Trypanosomes (family Trypanosomatidae, order Kinetoplastida) are unicellular flagellates. African trypanosomes are important as parasitic protozoa of humans and animals. *Trypanosoma brucei* causes sleeping sickness in humans, whereas *T. congolense* is the causative agent responsible for Nagana (animal African trypanosomiasis) in cattle. Trypanosomes have a complicated life cycle, living and multiplying freely in the blood and tissue fluids of their mammalian hosts. Trypanosomes are transmitted by Tsetse flies (genus *Glossina*), in which they undergo another developmental cycle. In Africa, over 50 million people live in areas with tsetse flies and trypanosomes. About 25,000 to 50,000 new infections of sleeping sickness are reported annually [1]. Nagana is a potential threat for 46 million cattle and it causes damage of about US $1,340 million per year [2]. If left untreated, trypanosome infections are fatal. Unfortunately, no vaccine is available against trypanosomes and chemotherapy still relies on drugs that were developed decades ago. Some of the drugs exhibit substantial toxic side effects [3]. Furthermore, drug resistance has been reported from animals [4]. Therefore, it is important to search for new drug candidates to treat sleeping sickness.

In an earlier publication we had analysed the cytotoxic potential of 34 alkaloids of various structural types against *T. brucei* and *T. congolense* [5]. Berbamine, berberine, cinchonidine, cinchonine, emetine, ergotamine, quinidine, quinine, and sanguinarine exhibited trypanocidal activities with ED50 values lower than 10 µM. These activities are similar to those of the antitrypanosomal drugs suramin and diminazene aceturate. The alkaloids also showed cytotoxicity against human HL60 cells [5]. In another study we could show that cell death in HL60 cells is due to apoptosis [6].

Many alkaloids, of which more than 21,000 structures have been found in plants, show a high degree of toxicity towards animals. Whereas many alkaloids are neurotoxins that interfere with neuroreceptors, ion channels or other parts of the neuronal signal chain, a substantial number of alkaloids are cytotoxic. Cytotoxicity is a result of the molecular interactions of an alkaloid with one or several important targets present in a cell. The main targets include DNA, RNA, and the associated enzymes and processes (i.e., replication, repair, transcription, DNA polymerase, RNA polymerase, reverse transcriptase, repair enzymes, topoisomerase, telomerase), protein biosynthesis, protein conformation, biomembrane integrity, and membrane proteins [for reviews see 7,8,9,10,11].

Cell biologists distinguish between necrotic and apoptotic cell death. If a cell is lysed by saponins or other detergents, or when it is mechanically wounded or exposed to physical stress (heat, freezing, hypoxia), then it dies quickly by necrosis and often causing an inflammation. Apoptosis or programmed cell death, that was discovered about 35 years ago in 1972 by Kerr, Wyllie, and Currie [12], is a central mechanism in the development of most organisms. Apoptosis is characterized by nuclear chromatin condensation, cytoplasmic shrinking, dilatation of endoplasmic reticulum, membrane blebbing, and the formation of apoptotic bodies. Programmed cell death does not cause inflammation as cells are digested by macrophages or neighboring cells. Apoptosis can be induced by many substances, among them several natural products, such as several alkaloids, polyphenols, terpenoids, or saponins that primarily interact with an important molecular target such as DNA, microtubules, biomembranes and receptors. Most of the anticancer drugs presently used in cancer therapy induce apoptosis [6,10].

Apoptosis can be induced by two pathways: the extrinsic pathway starts with an activation of death receptors on the cell surface, which leads to the activation of caspases. The intrinsic pathway is triggered by the permeabilization of mitochondrial membranes, furthermore by releasing cytochrome c and reducing ATP levels [13]. Cytochrome c and other apoptotic factors lead to the activation of caspase 9, which finally activates pro-caspase 3 to caspase-3 [14]. The activated caspases cleave cellular proteins (e.g. proteins of the cytoskeleton) and via caspase-activated DNAse (CAD) also chromatin. As a consequence, a typical sequence of morphological and biochemical degradation steps sets in. If the DNA of apoptotic human cells is being analysed by gel electrophoresis, a typical ladder pattern of fragmented chromosomes can be observed. The DNA fragmentation can be reliably monitored and quantified by flow cytometry.

We had found a pronounced cytotoxic effect of some alkaloids on trypanosomes [5]. Therefore, in view of the proapoptotic activity of alkaloids in human tumour cells [6, 10] the question to be answered in this communication was, whether programmed cell death (PCD) can be induced in trypanosomes and whether it is also responsible for cell death in *T. brucei* treated with cytotoxic alkaloids. For comparison we have analysed the initiation of apoptosis by the same alkaloids in a human leukemia cell line, Jurkat APO-S, which expresses CD95.

## Results and Discussion

### Induction of apoptosis in trypanosomes and leukemia cells (Jurkat APO-S)

We have selected a series of alkaloids belonging to several structural types. Most of them had been studied before and their cytotoxicity in trypanosomes and human HL60 cells had been determined [5, 6]. In this project, we have used a strain of *T. b. brucei* TC221 and Jurkat APO-S human leukemia cells for comparison, in order to find out whether cytotoxicity is due to apoptosis.

In a first set of experiments, known substances that can induce apoptosis, such as staurosporine and those that can change the membrane potential of mitochondria, such as carbonylcyanide M-chlorophenyl hydrazone (CCCP) and valinomycin, were studied as positive controls. Staurosporine induced a significant DNA fragmentation in HL60 cells [6] and in trypanosomes (Table 1). As can be seen from Table 1, CCCP and valinomycin significantly induced a change in membrane potential, both in Jurkat cells and in trypanosomes. These results indicate that programmed cell death can be initiated also in trypanosomes.

The main experiment included 55 alkaloids that were tested for their ability to induce DNA fragmentation in trypanosomes (Table 1). If an alkaloid was active, we also studied its ability to induce changes in mitochondrial membrane potential. In addition, we tested Jurkat APO-S cells to see whether the effects were similar in leukemia cells as in trypanosomes. About 28 alkaloids induced apoptosis at a concentration of 100 µM in *T. brucei.* For 14 alkaloids the EC_50_ values were below 100 µM: quinine, berberine, chelerythrine, emetine, sanguinarine, ajmalicine, ergotamine, harmine, vinblastine, vincristine, colchicine, chaconine, demissidine and veratridine. Especially active in *T. brucei* (EC_50_ below 10 µM) were chelerythrine, emetine, sanguinarine, and chaconine (Table 2). These alkaloids apparently induce PCD in trypanosomes.

**Table 1:** Induction of apoptosis in *Trypanosoma b. brucei* and in Jurkat APO-S cells.

Alkaloid (highest concentration tested)	Membrane depolarization		PCD/Apoptosis (DNA fragmentation)		
	Jurkat APO-S	*T. b. brucei*	Jurkat APO-S	Jurkat + z-VAD-fmk	*T. b. brucei*
**Quinoline alkaloids**					
Cinchonidine (10^-3^ M)	na	50%	45%	25%	EC_50_: 0.42 mM
Cinchonine (10^-4^ M)	na	na	na	nd	30%
Quinidine (10^-4^ M)	na	na	na	nd	45%
Quinine (10^-3^ M)	15%	50%	45%	45%	EC_50_: 59.9 µM
**Isoquinoline alkaloids**					
α-Allocryptopine (10^-4^ M)	na	na	na	nd	17%
Aporphine (10^-4^ M)	nd	na	nd	nd	na
Berberine (10^-4^ M)	15%	35%	na	na	EC_50_: 99.5 µM
Boldine (10^-4^ M)	nd	na	nd	nd	na
Chelerythrine (10^-4^ M)	100%	95%	35%	5%	EC_50_: 1.3 µM
Chelidonine (10^-4^ M)	na	na	30%	12%	na
Coralyne (10^-4^ M)	nd	na	nd	nd	na
Emetine (2.5 x 10^-4^ M)	5%	na	10^-5^ M 45%	10^-5^ M 16%	EC_50_: 9.8 µM
Laudanosine (10^-4^ M)	nd	nd	nd	nd	EC_50_: 0.5 mM
Noscapine (10^-3^ M)	nd	nd	nd	nd	EC_50_: 0.54 mM
Papaverine (10^-4^ M)	na	30%	18%	6%	10%
Protopine (10^-4^ M)	na	na	10%	4%	30%
Sanguinarine (10^-5^ M)	65%	50%	5%	5%	EC_50_: 4.8 µM
**Indole alkaloids**					
Ajmalicine (10^-4^ M)	nd	nd	nd	nd	50%
Ajmaline (10^-4^ M)	nd	nd	nd	nd	na
Brucine (10^-4^ M)	nd	nd	nd	nd	na
Ellipticine (10^-4^ M)	100%	90%	15%	10%	10%
Ergotamine (10^-4^ M)	nd	nd	nd	nd	EC_50_: 64 µM
Gramine (10^-4^ M)	nd	nd	nd	nd	na
Harmaline (10^-4^ M)	nd	nd	nd	nd	45%
Harman (10^-4^ M)	nd	nd	nd	nd	na
Harmine (10^-4^ M)	5%	30%	30%	10%	EC_50_: 74 µM
Physostigmine (10^-3^ M)	nd	nd	nd	nd	EC_50_: 0.54 mM
Strychnine (10^-5^ M)	nd	nd	nd	nd	na
Vinblastine (10^-5^ M)	na	nd	35%	15%	40%
Vincamine (10^-4^ M)	na	nd	na	na	na
Vincristine (10^-5^ M)	na	nd	40%	20%	35%
Yohimbine (10^-4^ M)	nd	nd	nd	nd	na
**Steroidal alkaloids**					
α-Chaconine (10^-5^ M)	nd	nd	nd	nd	EC_50_: 2.4 µM
Demissidine (10^-4^ M)	nd	nd	nd	nd	EC_50_: 14 µM
α-Solanine (10^-5^ M)	na	na	na	nd	EC_50_: 8.5 mM
Veratridine (10^-4^ M)	nd	nd	nd	nd	EC_50_: 43 µM
**Piperidine alkaloids**					
Arecoline (10^-3^ M)	nd	nd	nd	nd	45%
Coniine (10^-4^ M)	nd	nd	nd	nd	na
Lobeline (10^-4^ M)	nd	nd	nd	nd	na
Piperine (10^-3^ M)	50%	40%	30%	5%	EC_50_: 0.57 mM
Pseudopellerine (10^-3^ M)	nd	nd	nd	nd	na
**Purine alkaloids**					
Caffeine (10^-4^ M)	nd	nd	nd	nd	na
Theobromine (10^-4^ M)	nd	nd	nd	nd	na
Theophylline (10^-4^ M)	nd	nd	nd	nd	na
**Tropane alkaloids**					
Hyoscyamine (10^-3^ M)	nd	nd	nd	nd	32%
Methylscopolamine (10^-3^ M)	nd	nd	nd	nd	na
Tropine (10^-3^ M)	nd	nd	nd	nd	na
**Quinolizidine alkaloids**					
Cytisine (10^-4^ M)	nd	nd	nd	nd	na
Lupanine (10^-3^ M)	nd	nd	nd	nd	na
Sparteine (10^-3^ M)	nd	nd	nd	nd	na
**Other alkaloids**					
Aconitine (10^-5^ M)	nd	nd	nd	nd	na
Capsaicine (10^-5^ M)	nd	nd	nd	nd	na
Colchicine (10^-4^ M)	na	40%	50%	22%	50%
Ephedrine (10^-3^ M)	nd	nd	nd	nd	na
Nicotine (10^-3^ M)	nd	nd	nd	nd	na
**Controls**					
Actinomycin D (10^-3^ M)	nd	nd	35%	12%	nd
CCCP (10^-5^ M)	60%	50%	nd	nd	EC_50_: 30 µM
Staurosporine (5 x 10^-5^ M)	nd	nd	nd	nd	EC_50_: 29 nM
Valinomycin (10^-5^ M)	40%	100%	55%	40%	40%

DNA fragmentation and the change in membrane potential of mitochondrial membranes were determined as a measure for PCD and apoptosis. For alkaloids, which induce apoptosis in human leukaemia cells, we have assayed the participation of caspase 3 by applying the caspase 3 inhibitor z-VAD-fmk (trypanosomes do not have caspases); na = not active; nd = not determined.

Not all alkaloids induced PCD; most tropane, quinolizidine, piperine, pyridine, purine, steroidal, and diterpene alkaloids were not active up to a concentration of 100 µM (Table 1 and [6]). In general, alkaloids, which were found to be highly cytotoxic for trypanosomes also induced significant PCD (Table 2) in trypanosomes and in Jurkat cells. Some alkaloids, such as sanguinarine or emetine, showed a similar high toxicity in trypanosomes as in human leukemia cells, which would make them rather unsuitable for clinical applications. In other instances – quinine, ergotamine, berberine, cinchonidine, quinidine and cinchonine – the toxicity was substantially higher in trypanosomes than in leukemia cells. These alkaloids represent interesting lead structures for potential antitrypanosomal drugs, but we need further *in vivo* experiments showing their bioavailability. Most drugs, which are presently in use, such as suramin, are effective on blood stage forms but not active against brain infections. There is pharmacological evidence that the Cinchona alkaloids and ergot alkaloids can cross the blood brain barrier [8]. Interestingly, the Cinchona-alkaloids quinine have been used successfully against malaria parasites (*Plasmodium*) [3,7,23].

**Table 2:** Comparison between *T. brucei* and human leukemia cells (Jurkat APO-S, HL60). Data for cytotoxicity for *T. brucei* came from [5], those for HL60 from [5,6].

Alkaloid	Induction of apoptosis/PCD			Cytotoxicity	
	*T. brucei*	Jurkat APO-S 100 µM	HL60 MAC	*T. brucei*	HL60
Chelerythrine	EC_50_: 1.3 µM	+++	1 µM	nd	nd
Chaconine	EC_50_: 2.4 µM	nd	na	nd	nd
Sanguinarine	EC_50_: 4.8 µM	+	5 µM	EC_50_: 1.9 µM	EC_50_: 1.4 µM
Emetine	EC_50_: 9.8 µM	+++	0.5 µM	EC_50_: 0.04 µM	EC_50_: 0.09 µM
Demissidine	EC_50_: 14 µM	nd	nd	nd	nd
Veratridine	EC_50_: 43 µM	nd	na	nd	nd
Quinine	EC_50_: 59.9 µM	++	500 µM	EC_50_: 4.9 µM	EC_50_: 126 µM
Ergotamine	EC_50_: 64 µM	nd	50 µM	EC_50_: 3.2 µM	EC_50_: 32 µM
Harmine	EC_50_: 74 µM	++	100 µM	nd	nd
Berberine	EC_50_: 99.5 µM	na	100 µM	EC_50_: 0.53 µM	EC_50_: 27 µM
Cinchonidine	EC_50_: 420 µM	++	100 µM	EC_50_: 7.1 µM	EC_50_: 169 µM
Laudanosine	EC_50_: 500 µM	nd	na	nd	nd
Noscapine	EC_50_: 540 µM	nd	50 µM	nd	nd
Piperine	EC_50_: 570 µM	++	100 µM	EC_50_: >100 µM	nd
Vinblastine	10^-5^ M: 40%	+++	5 nM	nd	nd
Vincristine	10^-5^ M: 35%	+++	5 nM	nd	nd
Colchicine	10^-4^ M: 50%	+++	0.1 µM	EC_50_: 21 µM	nd
Ajmalicine	10^-4^ M: 50%	nd	50 µM	nd	nd
Harmaline	10^-4^ M: 45%	nd	na	EC_50_: 30 µM	nd
Quinidine	10^-4^ M: 45%	na	na	EC_50_: 0.7 µM	EC_50_: 400 µM
Cinchonine	10^-4^ M: 30%	na	na	EC_50_: 1.2 µM	EC_50_: 382 µM
Ellipticine	10^-4^ M: 10%	++	5 µM	nd	nd

Jurkat cells: += <10% apoptosis at 10^-4^ M; ++ = 10-30%, +++ = >30%. MAC = minimal apoptotic concentration

The active alkaloids have in common that they affect central molecular targets in cells (as shown in previous studies [7,8,9,10]): they intercalate DNA, and in consequence inhibit DNA and RNA polymerase, topoisomerases, and even ribosomal protein biosynthesis, or bind to tubulin/microtubules, thus acting as spindle poisons or to disturb membrane integrity (Table 3).

**Table 3:** Molecular targets of apoptotic alkaloids [7,8,9,10,15].

Alkaloid	Disturbance of Membrane fluidity	DNAintercalation	Protein biosynthesis inhibition	Microtubul inhibition	Neurotoxins
Berberine	na	++	++	na	+
Chaconine	+++	nd	+	na	+
Cinchonidine	na	+	++	na	+
Cinchonine	na	+	+	na	+
Colchicine	+	nd	na	+++	+
Demissidine	+++	nd	nd	nd	+
Emetine	na	+	++++	na	+
Ergotamine	na	++	+	nd	+++
Harmine	+	++	++	nd	+++
Quinidine	+	+	++	na	+
Quinine	na	+	++	na	+
Sanguinarine	+	+++	nd	na	++
Vinblastine	nd	++	nd	+++	+
Vincristine	nd	++	nd	+++	+

na = not active, nd = not determined

DNA damage, which can also be caused by intercalating compounds, alkylation or gamma-irradiation and other signals, can stimulate the tumor suppressor gene p53 in human cells. In consequence, Apaf-1 becomes activated, triggering apoptosis. P53 is a stress sensor that is modified by posttranslational modifications. Activated p53 reacts as a transcription factor for pro-apoptotic proteins, including BAX, and death receptors (CD95, TRAIL).

Some of the cytotoxic and PCD-inducing alkaloids (Table 1) had also been found to be trypanocidal in other studies, mainly against *T. cruzi*: steroidal alkaloids [20,32], piperine [21,33], cinchonidine [22], cinchonine [23], berberine [24,25], emetine [25,26,27], sanguinarine [28], ellipticine [29], harmaline [25,30], harmine [24,25,28,30], vinblastine [31], vincristine [21], and colchicine [34].

Programmed cell death is apparently not restricted to metazoan animals, but can also occur in a related fashion in protozoa; it has been detected in *Trypanosoma cruzi* and *T. brucei* [16,17,35,36]. In *T. brucei* for example, prostaglandin D, which inhibits growth of bloodstream forms, induces PCD with characteristic apoptosis features (maintenance of plasma membrane integrity, exposure of phosphatidylserine, loss of mitochondrial membrane potential, condensation of nuclear chromatin and DNA fragmentation) [35]. However, protozoa do not have caspases, they apparently lack the receptors of corresponding signaling pathways and do not form an apoptosome; therefore, the classical apoptosis mechanism cannot work in protozoa [36]. Published data indicate that apoptosis may occur in human cells in the absence of caspases [37,38,39]. To our knowledge, our report is the first to show that several of the trypanocidal alkaloids induce apoptosis-like cell death in *T. brucei* (albeit via unknown mechanisms) When searching for new drugs with antitrypanosomal activities, those secondary metabolites with apoptotic properties in metazoan cells could therefore be of major interest [10].

## Experimental

### Cell line and culture conditions

Jurkat APO-S derives from a human T cell leukemia; it is sensitive to apoptosis through CD95 expression. Jurkat-APO-S cells were maintained in RPMI 1640 medium without phenol red and L-glutamine (Gibco BRL, Life Technologies GmbH, Karlsruhe, Germany). The medium was supplemented with 10% inactivated fetal calf serum (Seromed®, Biochrom KG, Berlin, Germany), 100 units/ml penicillin, 100 µg/mL streptomycin (BioWhittaker, Walkersville, MD, USA) and 1% L-glutamine. Suspension-cultured cells were grown in 25 mL or 75 mL culture flasks (Cellstar, Greiner Labortechnik GmbH, Frickenhausen, Germany), incubated at 37 °C with 5% CO_2_ and diluted every 2 - 3 days to a final concentration of ca. 1 x 10^5^ cells/mL. Cell counts were performed with a Neubauer-count chamber, and general viability was assessed by Trypan blue exclusion.

### Culture of Trypanosoma b. brucei

*Trypanosoma b. brucei* TC221 (which came from D. Steverding, Hygiene-Institut, Heidelberg University) were cultivated in culture flasks with Baltz medium [18] at 37 °C and 5% CO_2_. Blood stream forms multiply ten-fold in suspension culture every 25 h, cultures were diluted every 2 to 3 days about 100-fold with fresh medium. A maximal density of 2 x 10^6^ cells was not exceeded.

### Tested substances

Alkaloids were either obtained commercially or had been isolated in our laboratory. There purity was usually higher than 98%. The origin of tested compounds has been documented in [5,6,15]. Compounds were dissolved in *aqua bidest*, DMSO or ethanol to final concentrations of 10^-1^ to 10^-3^ M. After a 1:10-dilution with RPMI medium the mixture was sterilized by filtration (0.2 µm diameter). Further 1:10-dilutions were made with sterile medium containing 10% of the solvent agent to ensure an equal amount of the solvent in the tests, about 1%.

### Induction of apoptosis

In order to induce apoptosis in Jurkat cells were seeded into 24-well plates (Gibco BRL), each well was filled with cell culture medium (1 mL). The cell number used in each experiment depended on the incubation time. Cells or trypanosomes were seeded into the wells with a concentration of 5 x 10^5^ or 1-2 x 10^5^ cells/mL, respectively. Compounds were given to the cells in various concentrations, with 10 to 5 µL/well. Untreated cultures and cultures treated with medium containing 10% solvent were used for control measurements. The caspase-3 inhibitor z-VAD-fmk was added 30 min before the addition of test compounds. Experiments were run for 24 h.

### Analysis of cell death

Apoptosis was examined by cell morphology (fluorescence microscopy), DNA gel electrophoresis, but mainly by flow cytometry. Also the activity of caspase 3 (including the caspase 3 inhibitor z-VAD-fmk) and membrane potential were determined using actinomycin D, CCCP (carbonylcyanide M-chlorophenyl hydrazone), staurosporine, and valinomycin as positive controls.

### Flow cytometry analysis:

Treated or untreated cell culture (1 mL) was harvested from the 24-well plates and centrifuged for 10 min at 4 °C and 400 x *g*. After a washing step with HBSS (Hank’s balanced salt solution without Mg^2+^/Ca^2+^, 1 mL, Gibco BRL) cells were incubated with HBSS (900 µL) and PC-buffer (Na_2_HPO_4_ 0.2 M, citric acid 0.1 M, pH 7.8, 300 µL) for 5 min, then centrifuged as described above. Cells were fixed with 1 ml ice-cold 70% ethanol and incubated at –20 °C for at least 2 h. Apoptotic cells were detected using propidium iodide (PI) staining [19]. Cells in 70% ethanol were centrifuged for 10 min at 4 °C and 400 x *g*, washed once with HBSS (1 mL), and incubated with PI staining solution (containing 0.05 mg PI/ml HBSS and 0.5 mg RNAse/mL HBSS, 200 µL) for at least 30 min before measurement with the FACScan analyzer. Experiments were performed in triplicate and repeated at least three times.

### Determination of mitochondrial membrane potential

An early step in the intrinsic apoptotic cascade is a reduction of the mitochondrial membrane potential. The change in membrane potential can be determined using a fluorescent dye, JC-1. If the membrane potential is between –170 and -220 mV, than JC-1 produces a red fluorescence. If the potential is lowered a green fluorescence can be detected. These changes can be recorded by the FACSan analyzer. The membrane potential was determined, 2, 4, 6 and 8 h after the application of the alkaloids. Treated or untreated cell culture (1 mL) was harvested from the 24-well plates and centrifuged for 10 min at 4 °C and 400 x *g* and washed with HBSS (without Ca^2+^, Mg^2+^, phenol red, 500 µL). The sediment was stained with JC-1 (1 µg/mL). After 15 min incubation in the dark a 37 °C, HBSS (400 µL) was added and the culture subjected to FACS measurements, by recording red or green fluorescence. Experiments were performed in triplicate and repeated at least three times.

### Data analysis

A total of 10,000 cells per sample was analyzed by FACScan and Cell Quest software (Becton Dickinson, Heidelberg, Germany) and with WinMDI software (version 2.7, Microsoft Corp.). SigmaPlot 8.0 was used to calculate EC_50_ values.
